# An update on gastrointestinal nematodes in reindeer (*Rangifer tarandus tarandus*) in Iceland

**DOI:** 10.1016/j.ijppaw.2025.101163

**Published:** 2025-11-17

**Authors:** R.K. Davidson, S. Dembereldagva, I.H. Nymo, T. Mørk, J. Sánchez Romano, R. þórarinsdóttir, K.S. Utaaker, S.G. þórisson, M. Tryland

**Affiliations:** a Norwegian Veterinary Institute, Holtvegen 66, 9016, Tromsø, Norway; b Department of Arctic and Marine Biology, UiT The Arctic University of Norway, Tromsø, Norway; c Erdenedrilling LLC, Ulaanbaatar, Mongolia; d Institute of Zoology, Zoological Society of London, London, United Kingdom; e East Iceland Nature Research Centre, Tjarnarbraut 39b, Egilsstaðir, 700, Iceland; f Northeast Iceland Nature Research Centre, Hafnarstétt 3, Húsavík, 640, Iceland; g Faculty of Bioscience and Aquaculture, Nord University, Bodø, N-8026, Norway; h Norwegian Veterinary Institute, P.O. Box 64, Ås, N-1431, Norway; i Department of Forestry and Wildlife Management, University of Inland Norway, Koppang, Norway

**Keywords:** Morphology, McMaster, Abomasum, Baermann, Spill-over, Sheep, Wild reindeer

## Abstract

Eurasian tundra reindeer (*Rangifer tarandus*) were introduced to Iceland from Norway in the late 18th century and have thrived in Eastern Iceland. In 2003–2005 the parasitic fauna was studied, and Icelandic reindeer were found to lack many parasites common to Norwegian reindeer. This study from 2018 provides an updated comparison. Abomasal content and faeces were collected from the 117 reindeer (63 adults, 22 yearlings, 17 calves, 15 age not recorded), from: 1 - West (N = 29), 2 - Central (N = 44), 3–9 - East (N = 40), not recorded (N = 4) management areas hunted in 2018. Not all animals were examined by all methods. Abomasal nematode counts (N = 81) were carried out in addition to faecal egg and larval counts, using modified McMaster (N = 111) and Baermann (N = 108). Abomasal nematodes were detected in 31 % of samples, with low mean abundance (48) and intensity (160). Males had higher prevalence (46 %) and mean abundance (89) than females (24 %; 29). The sheep gastrointestinal nematode (GIN) *Teladorsagia circumcincta* predominated, although, for the first time, single specimens of *Spiculopteragia boehmi (Gebauer,1932)* and a male nematode with morphology suggestive of *Ostertagia arctica*, a minor morph of *O. gruehneri*, were detected. *Trichostrongylus axei* was not detected. Trichostrongylidae and *Aonchotheca* egg prevalence was 35 % (mean abundance eggs per gram, EPG, 12, mean intensity 33 EPG) and 23 % (mean abundance 8 EPG; mean intensity 34 EPG) respectively. No faecal larvae were detected. There were geographic as well as sex related differences in abundance. Trichostrongylidae eggs prevalence, but not abundance, was higher in 2018 compared to 2005, and an opposite trend with abomasal nematode counts was seen. Reindeer in Iceland still have a low prevalence and abundance of GINs, dominated by *T. circumcincta*. The monitoring of GIN in this population provides a simple means of evaluating population health in a time with changing climate.

## Highlights

• Low prevalence and intensity of gastrointestinal nematodes in Icelandic reindeer.
• Sheep nematode *Teladorsagia circumcincta* predominates.
• *Spiculopteragia boehmi* detected for the first time in this population.
• Region and sex explained some of the variation in prevalence and abundance.

## Graphical abstract

**Figure:**
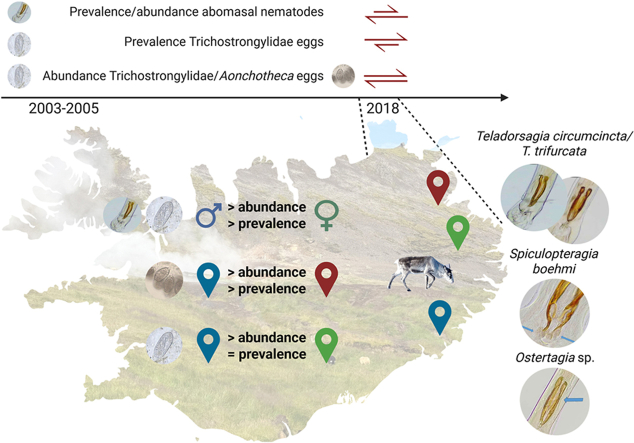

## 1 Introduction

### 1.1 Reindeer in Iceland

Semi-domesticated Eurasian tundra reindeer (*Rangifer tarandus*) were imported to Iceland from Kautokeino in Finnmark county, Norway, in the late 18th century and established themselves as a feral population in the north-eastern part of the country (þórisson, 1984). Given that reindeer have no natural predators in Iceland, the population has been divided into nine management areas which are managed by annual hunting quotas. The aim of the hunt is to ensure the population remains sustainable, to limit winter population densities at one animal per square kilometre of suitable pastures (all vegetated land) and to maintain a sex ratio of six males to ten females (þórisson, 2018). The winter population (after the hunt and before calving) has grown from 35 reindeer imported in 1787 to close to 5000 in 2010–2019 (Helgason and Ágústsdóttir, 2023; þórisson, 1984, 2018). The population size has decreased the last few years (þórisson et al., 2021, þórisson et al., 2022; þórisson and þórarinsdóttir, 2022), probably as a result of high harvest pressure in some management areas, caused by prior overestimation of population sizes. The population is currently thought to number around 3500–4000 individuals (personal communication Hálfdán Helgi Helgason, 2025). There have been no further reindeer imports or contact with other reindeer populations. The reindeer populations in Eastern Iceland share grazing with sheep and horses. It is estimated that around 100 000 sheep graze the eastern part of Iceland (Árnason et al., 2022; Helgason and Ágústsdóttir, 2023) with large local variations in sheep density.

### 1.2 Previous parasitological surveys of this population

In 2003–2005, Guðmundsdóttir (2006) investigated the parasitic fauna of Icelandic reindeer and identified 17 species of parasites including eight protozoan, five nematode and one cestode species (Table 1). The study identified two new species of *Eimeria: Eimeria rangiferis* and *Eimeria hreindyria*, in addition to *Eimeria mayeri* which had been previously identified (Guðmundsdóttir and Skírnisson, 2005, 2006). Guðmundsdóttir (2006) also documented infections with *Entamoeba* sp., *Giardia duodenalis*, *Sarcocystis* sp., *Ostertagia ostertagi*, *Teladorsagia circumcincta*, *Trichostrongylus axei*, and *Aonchotheca bovis*, as well as eggs from *Moniezia expansa* in one individual. In addition, Guðmundsdóttir (2006) identified three types of nematode eggs (Trichostrongylidae, *A. bovis* and *Nematodirus spathiger*) in reindeer calf faecal scats sampled during three different times in June–August 2003, as well as in adults sampled during the hunt (August–September) and later (November–March). Guðmundsdóttir concluded that bottlenecks during the establishment and expansion of the reindeer population in Iceland had resulted in a loss of a number of reindeer specific parasites like *Ostertagia gruehneri* and warble flies (*Hypoderma tarandi*) which are otherwise very common in reindeer populations across the Palearctic, including Norway (Dallas et al., 2000; Eira et al., 2025; Hoar et al., 2012; Idland et al., 2021; Jokelainen et al., 2019; Mørk et al., 2024). The prevalence and abundance (number of counts of a parasite in population as a whole, including non-infected individuals) (Bush et al., 1997) of gastrointestinal nematodes (GIN) in wild and semi-domesticated reindeer in Norway (Eira, 2022; Idland et al., 2021; Robertsen, 2020; Utaaker et al., 2023) is generally higher than in the population from Iceland (Table 1) (Guðmundsdóttir, 2006).

**Table 1:** Prevalence and mean abundance (eggs per gram - EPG) of Trichostrongylidae and *Aonchotheca* sp. in faecal samples from Eurasian tundra reindeer (*Rangifer tarandus*) from different regions and years in Iceland and Norway.

Location	Management area/Herd	Study	Age class	Sampling months	Year	N	Trichostrongylidae eggs		*Aonchotheca* sp. Eggs		Other nematode eggs identified
							Prevalence [95 % CI]	Mean abundance EPG [95 % CI]	Prevalence [95 % CI]	Mean abundance EPG [95 % CI]	
**Iceland**	East, Central and West	This study	All	August–September	2018	111	35.1 [26.9–44.4]	11.8 [7.5–16.2]	22.5 [15.7–31.1]	7.7 [4.4–11]	
	East, Central and West	Guðmundsdóttir (2006)	All	June–March	2003–2005	246	16.3 [12.2–21.4]	18.5 [10.7–26.3]	12.2 [8.7–16.9]	9.1 [5.5–12.8]	*Nematodirus* spp.
**Semi-domesticated reindeer northern Norway**	Spierttagáisá, Finnmark	Idland et al. (2021)	All	October–January	2017 & 2018	46	84.8 [71.8–92.4]^a^	46	2.2 [0.4–11.3]	20	*Nematodirus* spp.
	Neiden, Finnmark		All	October–January	2017 & 2018	68	47.1 [35.7–58.8]	16	69.1 [57.4–78.8]^a^	37	*Nematodirus* spp.
	Karasjok Vestre/Kárášjoga Oarjjabealli, Finnmark	Robertsen (2020)	All	December	2018	19	63 [38–84]	82	26 [9–51]	26	
	Doukta, Nordland	Eira (2022)	All	December	2020	43	86.1 [75.7–96.4]^a^	53	44.2 [29.3–59]	58	*Nematodirus* spp.
**Wild reindeer southern Norway**	Knutshø	Utaaker et al. (2023)	All	August–September	2018 & 2019	126	91.2 [87–96.7]^a^	202	1.6 [0–3.9]	43	*Nematodirus* spp.; *N. battus*; *Trichuris* sp.; *Skrjabinema* spp.
	Forollhogna		All	August–September	2018 & 2019	102	95.2 [91.2–99.3]^a^	110	24.8 [16.5–33]	52	*Nematodirus* spp.; *N. battus*; *Trichuris* sp.; *Skrjabinema* spp.
	Nordfjella		All	August–September	2018 & 2019	55	75.5 [63.9–87.1]^a^	114	11.3 [2.8–19.9]	25	*Nematodirus* spp.; *N. battus*; *Trichuris* sp.; *Skrjabinema* spp.

a Significantly higher egg prevelance in these herds than in both studies from Iceland.

### 1.3 Study aims and objectives

The aim of our study was to update the baseline data on the prevalence and abundance of GIN including abomasal nematode diversity in Icelandic reindeer and compare our results with previous findings.

## 2 Material and methods

### 2.1 Sampling of reindeer

**Fig. 1:**
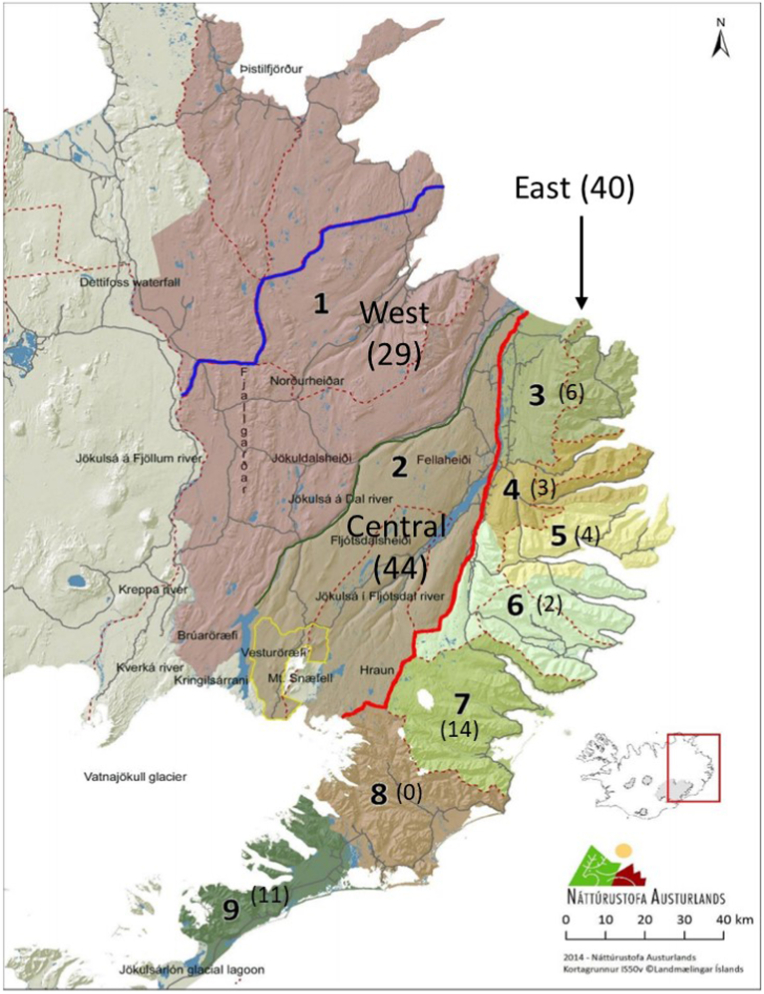
A map of eastern Iceland, showing the nine different reindeer management areas, adapted from þórisson (2018). The red line shows the limit of the highland plateau, which covers management area 1 (West) and management area 2 (Central), and management areas 3–9 (East). The number of reindeer examined from each management area are shown in brackets. The blue line shows the previous northern border of area 1 (2012–2014), the green the division between areas 1 and 2. No reindeer were investigated from management area 8.

Abomasal contents and faeces were collected from hunted reindeer (N = 117) at the meat processing facilities serving eight of the nine management areas in Iceland, during 24th August - September 8, 2018 (Fig. 1) by qualified staff. All, except 16, of the animals were sampled on the same day as being shot by hunters. Fourteen animals were sampled one day after being shot whilst a further two were sampled within two days of being shot. This gave a mean time between death and sampling of 0.21 days.

The abomasum was cut along the greater curvature, and the contents collected in a bucket (Eysker and Kooyman, 1993). The mucosa was then washed and the wash water collected in the bucket and filled to the 2 L mark. The contents were homogenised and a 100 mL subsample was taken and frozen to −20 °C whilst faeces was taken per rectum and stored refrigerated (4–6 °C), until shipping to Norway for further analysis. The reindeer age class (adult >2 years, yearling 1–2 years, calf <1 year old), sex (male or female) and geographic origin (management area 1–9) were recorded when possible (Table 2).

**Table 2:** The number of reindeer (*Rangifer tarandus*) sampled for faecal and abomasal parasite analysis during the 2018 hunting season (24th August-8th September) in Iceland by age class, sex and grouped management area. Not all reindeer had faecal and/or abomasal samples.

Age class	Sex	Grouped management area				*Total*
		East	Central	West	Not registered	
**Calf (<1 year)**	**Male**	2	4	1	2	*17*
	**Female**	1	4			
	**Not registered**	2			1	
**Yearling (1–2 years)**	**Male**	2	3	1		*22*
	**Female**	4	7	5		
**Adult (>2 years)**	**Male**	14	1	7		*63*
	**Female**	9	22	10		
**Not registered**	**Male**				1	*15*
	**Female**	6	3	5		
***Total***		*40*	*44*	*29*	*4*	*117*

Once sampling was completed the samples were shipped to Norway in an isopore box with frozen cooler elements, ensuring that the faeces did not have direct contact with the frozen samples or cooler elements. The time from sampling in Iceland until analysis in the lab in Tromsø varied from 7 to 19 days (mean 10 days) for faecal egg and larval counts. Many of the abomasal samples arrived defrosted and had to be refrozen upon arrival.

### 2.2 Parasitological analyses

Not all samples were analysed with all methods: abomasal nematode counts N = 81, faecal egg counts N = 111, and faecal larval counts N = 108. The geographic origin data was grouped: West = management area 1 (N = 29), Central = management area 2 (N = 44) and East (coastal) = management areas 3–9 (N = 40) in addition there were four samples in which the management area was not recorded (Table 2, Fig. 1).

The frozen abomasal samples were thawed individually, two to four samples per day, and analysed under a stereomicroscope (Leica MZ16 with a CLS150× lightsource) at low-level magnification (up to 40x) with direct light. Nematodes were counted, separated by sex and stored refrigerated in 70 % ethanol until species identification of the male nematodes. Species identification was based upon the morphological characteristics of the male nematodes (spicule form, length, dorsal ray pattern, genital cone and bursa morphology) after clearing in polyvinyl lactophenol (Waldeck, Münster, Germany) for a few minutes using bright-field microscopy (Leica microsystems DM750, at 100 times magnification) to visualise the bursa, spicules and oesophageal valve length. Pictures were taken using a Leica ICC50W camera attached to the microscope. The species were identified for both major and minor morphotypes (Dróżdż, 1965, 1995; Fruetel and Lankester, 1989; Umur et al., 2011). The abomasal samples represented 5 % of the total abomasal content collected. The total abomasal nematode count was therefore based on the total number of nematodes identified x20. The individual parasite diversity estimates were based on determination of the different types of faecal eggs in combination with the identified male abomasal nematodes.

The faecal samples were analysed for egg counts using a modified McMasters method (using 3 g of faeces), and, when sufficient faeces was present (>8 g), for larval counts using the Baermann's technique (Taylor et al., 2016).

### 2.3 Statistical analyses

Summary statistics and modelling were done using JMP 18.2.1 (JMP statistical discovery, SAS) and trends in parasite prevalence related to reindeer age class, sex and grouped management areas were evaluated using contingency analysis and Fischer's exact tests (both two-sided and one-sided). The 95 % confidence intervals (CIs) were calculated for parasite prevalence, mean egg (EPG) abundance and intensity as well as for abomasal parasite counts. Trends relating to parasite abundance and intensity (mean and median EPG, abomasal nematode counts) by age class, sex and grouped management area were tested using ANOVA and Wilcoxon's test for ranked nonparametric comparisons. A general linear model (binomial distribution, maximum likelihood method with logit function) was made to investigate trends in prevalence between reindeer age class, sex and grouped management area. A significance level of p = 0.05 was selected for all analyses.

## 3 Results

### 3.1 Abomasal nematodes – prevalence and abundance

Adult nematodes were detected in 24 of the 81 (29.6 %, 95 % CI [20.8–40.3]) abomasal content samples examined. Contingency analysis found no significant difference in prevalence by reindeer age class (p = 0.45) or geographic origin (p = 0.22). However, reindeer males had significantly higher abomasal nematode prevalence (p = 0.05) than females (Table 3).

**Table 3:** The prevalence with 95 % confidence interval (CI), mean parasite abundance and intensity (with 95 % CI) and range as measured by faecal egg counts (FEC) as eggs per gram (EPG) faeces for Trichostrongylidae and *Aonchotheca* sp. Eggs as well as total abomasal nematode counts (Abo) from Eurasian tundra reindeer (*Rangifer tarandus*) hunted in Iceland between 24th August - September 8, 2018.

Classification		N FEC/Abo	Trichostrongylidae egg Prevalence Mean abundance [EPG CI] *Mean intensity [EPG CI] EPG Range*	*Aonchotheca* sp*.* Egg Prevalence Mean abundance [EPG CI] *Mean intensity [EPG CI] EPG Range*	Abomasal count Prevalence Mean abundance [CI] *Mean intensity [CI]* *Abo Range*
**Sex**	**Male**	37/26	**56.8 % [40.9-71.3]**^a^ 19.7 [12.0–27.3]^a^ *33.8 [24.9-42.6]* ***0–74***	**29.7 % [17.5-45.8]** 9.5 [3.7–15.3] *35.9 [20.8-50.9]* ***0–66***	**46.2 % [28.8-64.5]**^a^ 88.9 [3.9–173.8]^a^ *192.6 [14.3-370.8]* ***0–1000***
	**Female**	71/53	**23.9 % [15.5-35.0]**^a^ 8.0 [2.5–13.5]^a^ *33.0 [13.0-52.9]* ***0–181***	**19.7 % [12.1-30.4]** 7.1 [2.9–11.3] *32.0 [21.4-42.6]* ***0–100***	**24.1 % [14.6-36.9]**^a^ 29.2 [1.7–56.6]^a^ *118.9 [11.9-226.0]* ***0–620***

**Age class**	**Calf**	17/12	**41.2 % [21.6-64]** 12.8 [0.9–24.8] *31.1 [16.6-45.7]* ***0–60***	**5.9 % [10.5-27.0]** 1.2 [-7.5-9.8] *19.6* ***0–*20**	**41.7 % [19.3-68.0]** 28.3 [-58.0-114.6] *68 [5.7-130.3]* ***0–140***
	**Yearling**	21/19	**23.8 % [10.6-45.1]** 7.1 [-3.9-18.1] *28.4 [6.7-50.0]* ***0–59***	**33.3 % [17.2-54.6]** 8.8 [1.0–16.6] *26.3 [17.2-35.4]* ***0–42***	**21.1 % [8.5-43.3]** 5.8 [-62.8-74.4] *27.7 [-6.8-62.1]* ***0–60***
	**Adult**	59/43	**37.3 % [26.1-50]** 14.7 [8.2–21.1] *38.6 [22.9-54.4]* ***0–181***	**23.7 % [14.7-36.0]** 9.5 [4.8–14.2] *40.2 [25.0-55.4]* ***0–100***	**32.6 % [20.5-47.5]** 78.4 [32.8–124.0] *240.8 [78.9-402.6]* ***0–1000***

**Grouped management area**	**West**	28/26	**32.1 % [17.9-50.7]** 12.3 [4.1–20.5] *36.9 [21.7-52.0]* ***0–74***	**7.1 % [2.0-22.6]**^a^ 2.1 [-1.1-5.3]^a^^,^^c^ *29.6 [-95.8-155.1]* ***0–40***	**38.5 % [22.4-57.5]** 58.5 [1.1–115.8] *152.0 [7.44-296.6]* ***0–620***
	**Central**	41/34	**24.4 % [13.8**–**39.3]** 5.9 [2.1–9.8]^b^ *23.7 [14.7-32.7]* ***0–59***	**22.0 % [12.0**–**36.7]**^a^ 5.9 [2.0–9.7]^a^ *26.7 [18.8-34.6]* ***0–42***	**20.0 % [10.0**–**35.9]** 21.8 [-0.7-44.2] *105.7 [2.9-208.5]* ***0–340***
	**East**	38/19	**47.4 % [32.4**–**62.7]** 18.4 [7.8–29.1]^b^ *38.9 [20.1-57.8]* ***0–181***	**34.2 % [21.2**–**50.1]**^a^ 13.3 [5.3–21.4]^a^^,^^c^ *38.9 [22.1-55.7]* ***0–100***	**36.9 % [19.1**–**59.0]** 81.9 [-31.1-195.0] *222.4 [-109.4-554.2]* ***0–1000***

**Total**	**All**	111/81	**35.1 % [26.9**–**44.4]** 11.8 [7.4–16.3] *33.0 [23.4-42.4]* ***0–181***	**22.5 % [15.7**–**31.1]** 7.7 [4.4–11.0] *34.2 [25.2-43.2]* ***0–100***	**30.9 % [21.9**–**41.6]** 48.2 [15.7–80.7] *154.3 [59.1-249.5]* ***0–1000***

a Significant difference within classification group (sex; age class or grouped management area).

b The Trichostrongylidae abundance EPG was significantly higher in East than the Central management areas with both student t-test and nonparametric analysis (p < 0.05).

c The *Aonchotheca* abundance EPG was significantly higher in the East than the West management areas with student t-test (p < 0.01) and nonparametric analysis (p < 0.03).

Mean abomasal nematode abundance was 49.1 (95 % CI for the mean 16.2–82.1, median 0, range 0–1000). No significant differences were seen by reindeer age class (p = 0.18), sex (p = 0.08) or geographic origin (p = 0.33) in ANOVA analysis. Nonparametric analysis of ranked abomasal nematode counts showed that reindeer males had significantly higher abomasal nematode abundance (counts) than females (p = 0.02). Neither geographic origin (p = 0.30) nor reindeer age class (p = 0.32) were significantly different in ranked nonparametric tests (Table 3). The mean intensity of abomasal nematodes was 160 (95 % CI for the mean 62.6–254.4, median 60, range 20–1000). Males had higher abomasal intensity (mean 192.6 [95 % CI 14.3–370.8]; median 60; range 20–1000) than females (mean 118.9 [95 % CI 11.9–226.0]; median 40; range 20–620) but this was not significant in ANOVA (p = 0.44) or nonparametric analyses (p = 0.16). Nor were there any significant differences in abomasal parasite intensity between geographic origin (p = 0.66) or age class (p = 0.17)-

The total number of abomasal nemtaodes (abomasal counts) explained 13 % of the variation in Trichostrongylidae egg abundance with bivariate fit (p < 0.001) showing an increasing trend of egg abundance with higher abomasal counts. Comparison of abomasal counts and egg abundance by sex showed significant correlation for males (p = 0.03) but not females (p = 0.98). Ten reindeer, which had abomasal nematode counts ranging from 15 to 620 (mean 102; median 25), had no detectable Trichostrongylidae eggs on McMaster analysis.

### 3.2 McMaster and Baermann analysis – prevalence and abundance of nematode eggs and larvae

Reindeer with missing data for sex, age class or management area were excluded from the relevant classification category, but not from the dataset. The reindeer sex ratio in the sample was skewed between areas. Significantly more reindeer males came from the East area, whilst more females were from the Central area (p = 0.02). There were no significant regional differences in the age class of the reindeer nor different age class trends between the sexes.

Trichostrongylidae eggs were detected in 35 % of the samples (Table 1; Table 3). Males (N = 37) had a significantly higher Trichostrongylidae egg prevalence (p < 0.001) than females (N = 71). There were no significant differences in egg prevalence between reindeer age classes (p = 0.45) or grouped management areas (p = 0.09). Modelling showed that reindeer age class and sex were the best predictors for differences in Trichostrongylidae prevalence (Table 4). The abundance data for Trichostrongylidae eggs showed overdispersion, with most of the faecal samples having median egg counts (EPG) of zero - except in males, where higher counts were observed. The egg counts were low (Table 4) with female reindeer having significantly lower mean (p = 0.01) and median abundance (p < 0.01) than males. There were no significant differences in mean or median abundance (EPG) by reindeer age class (p = 0.5). There were no significant differences in mean abundance by grouped management area (p = 0.07) with ANOVA analysis, although nonparametric analysis found significantly higher Trichostrongylidae abundance in animals from the East compared to the Central area (p < 0.05). The mean intensity of infection (EPG) was low (mean EPG 33.7 [95 % CI 24.4–42.9]; median 19.9). There were no significant differences in mean EPG intensity by grouped management area (p = 0.41), sex (p = 0.94) or age class (p = 0.73) with ANOVA analysis.

**Table 4:** The top three General linear binomial models with logit function using maximum likelihood method to evaluate Trichostrongylidae and *Aonchotheca* sp. Egg prevalence in response to age class, sex and geographic origin *(grouped management area: East, Central, West)* of the reindeer investigated in Iceland (2018). Significant effects in the model are highlighted in bold.

Parasite egg prevalence	Effects	AICc	P-value whole model	Observations	Degree of freedom	Direction of effect
Trichostrongylidae	Age class; Management area; **Sex**; Management area∗Sex	122.3	0.05	92	7	Male > Female
	Age class; Management area; **Sex**	120.2	0.04	92	5	
	Age class; **Sex**	119.0	0.01	94	3	
*Aonchotheca* sp.	Age class; **Management area**; **Sex**; Management area∗Sex	103.6	0.03	92	7	East > Central + West
	Age class; **Management area**; Sex	102.8	0.04	92	5	
	Age class; **Management area**	102.1	0.03	94	4	

The only other nematode egg detected was *Aonchotheca* sp. (Table 3, Table 4) at a prevalence of 23 %. The mean abundance (7.7 EPG [95 % CI 4.4–11.0]; median 0) and intensity (34.2 EPG [95 % CI 25.2–43.2]; median 20.1) of *Aonchotheca* infection (EPG) was low. There were no significant reindeer age class or sex related differences in prevalence (p = 0.24 and p = 0.09), abundance (p = 0.24 and p = 0.5) or intensity (p = 0.35 and p = 0.67). There were, however, significant geographic differences in prevalence (p = 0.02) and abundance (p = 0.02), but not intensity (p = 0.44). Reindeer from the East management area had higher *Aonchotheca* prevalence and abundance than those from the West. Modelling showed that reindeer age class and geographic origin emerged as the best explanatory factors for differences in *Aonchotheca* prevalence.

No *Dictyocaulus* sp. or protostrongylidae larvae were detected (0 % [95 % CI 0–3.3]) using Baermann analysis.

### 3.3 Parasite diversity: faecal and abomasal parasites

In addition to *Aonchotheca* and Trichostrongylidae eggs, hatched Trichostrongylidae larvae (in six reindeer), *Eimeria* sp. (in two individuals) and *Moniezia expansa* (in one individual) were detected. Half of the individuals sampled (faeces and abomasal samples) had no parasites detected (55/111; 50 % [95 % CI 40–59]). Single-genus infections were seen in 40 reindeer (36 % [95 % CI 28–45]) with 25 having just Trichostrongylidae type eggs and 13 having only *Aonchotheca* eggs. Eleven reindeer had mixed infections with two species (10 % [95 % CI 6–17]); three with three species (3 % [95 % CI 1–8]), and one with four and five respectively (1 % [95 % CI 0–5]). Male reindeer had significantly greater parasite diversity than females (p < 0.01) with on average 1.2 [0.9–1.5] different parasite species (ranging from 0 to 5), compared to females which had on average 0.5 [0.3–0.7] species (ranging from 0 to 3). There were no age class related differences in parasite diversity (p = 0.71), but there were significant geographic differences (p = 0.01) with animals from the East having greater diversity (mean 1.05 [0.8–1.3]) than the Central (mean 0.6 [0.4–0.9]) and West (mean 0.4 [0.01–0.7]) management areas.

Abomasal nematodes were identified in 25 reindeer. However, it was only possible to identify these abomasal nematodes to species level in 14 of these reindeer (Table 5). Four nematode species were identified in reindeer abomasa, including *T. circumcincta* (including its minor morph *T. trifurcata)*, *S. boehmi*, and *Ostertagia* spp., with some limitations due to sample degradation. Notably, *S. boehmi* was recorded for the first time in this population, though methodological challenges and potential biases, such as lack of male specimens and differences in sampling methods, likely influenced parasite detection and diversity compared with the earlier study.

**Table 5:** Species of abomasal nematodes identified in the abomasal content of Eurasian tundra reindeer (*Rangifer tarandus*) (N = 81) in Iceland, analysed from the 2018 hunting season (24th August-8th September) by age class and sex. It was not possible to speciate the abomasal nematodes in 11 individuals given the presence of female nematodes only. These are shown as not identifiable.

Grouped management area	Prevalence % (no. Positive/no. examined)	Species abomasal nematode	Age class								Total all age classes
			Calf		Yearling		Adult		Age not registered		
			F	M	F	M	F	M	F	M	
East	36.9 (7/19)	*T. trifurcata + Ostertagia* sp.					1				*1*
		*Ostertagia* sp.						1			*1*
		*T. circumcincta*						1	1		*2*
		Not identifiable			1	1		1			*3*
Central	20.0 (7/35)	*T. circumcincta*	2				2				*4*
		Not identifiable	1	1		1					*3*
West	38.5 (10/26)	*Ostertagia* sp.						1			*1*
		*T. circumcincta*					2	1			*3*
		*T. circumcincta/T.trifurcata + O. arctica*						1			*1*
		*Not identifiable*			1		1	2	1		*5*
Not registered	100 (1/1)	*T. circumcincta*		1							*1*

**Total all regions**	*30.9 (25/81)*		*3*	*2*	*2*	*2*	*6*	*8*	*2*		*25*

**Fig. 2:**
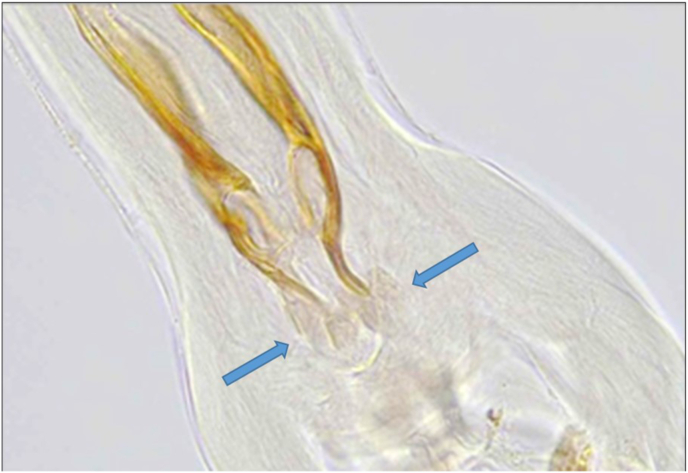
A photo of the spicules of *Spiculopteragia boehmi*, from the abomasal contents of an adult four-year-old male reindeer from the East management area (Botnsdalur) in Iceland, showing the characteristic fan shaped ends (blue arrows). Magnification ×200.

**Fig. 3:**
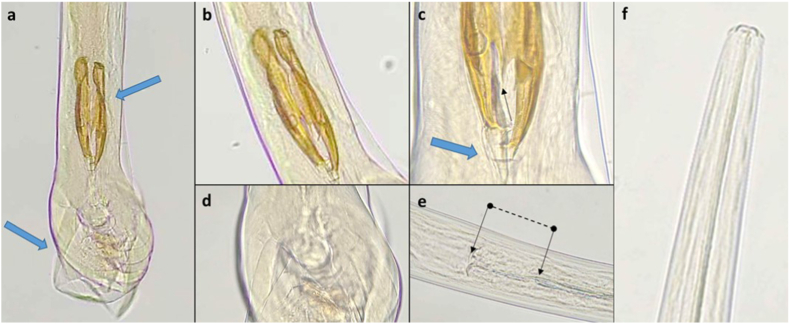
The single specimen of *Ostertagia* that was suggestive of *O. arctica* (the minor morphotype of *O. gruehneri*) based on spicule morphology and dorsal ray conformation (a–c), gubernaculum (c, blue arrow), dorsal lobe in ventral view (d), oesophageal valve proportions (e) and head shape (f) from the abomasal contents of an adult (3–5 year old) male reindeer hunted in the West management area (Kollseyra) in Iceland. Magnification ×200.

*Teladorsagia circumcincta*/*T. trifurcata* predominated and was found alone or in mixed infections in 13 reindeer from the East, Central and West management areas. *Spiculopteragia boehmi* was found in one adult male from the East (Botnsdalur, Fig. 2). This identification was based on the fan-shaped ends of the two spicules with similar lengths and varying width, the lack of a gubernaculum, and a 2-2-1 bursal ray pattern (Dróżdż, 1965; Umur et al., 2011). *Ostertagia* spp. were found in the West and East, but not the Central management areas. A single *Ostertagia*, tentatively identified as *O. arctica* (the minor morphotype o*f O. gruehneri*), was found in one adult male from the West (Kollseyra, Fig. 3). This identification was based on the presence of cuticular ridges, bursal rays with a 2-1-2 configuration, spicule morphology including trifurcation in the distal third, a sharp point on the shorter dorsal process and the longer ventral process ending in a sharp tip, as well as the presence of a gubernaculum (Dróżdż, 1965, 1995; Fruetel and Lankester, 1989; Lichtenfels et al., 1990). Further investigation of the accessory bursal membrane, Sjöbergs organ and genital cone although not optimal was also suggestive of *O. arctica*. It was not possible to identify the species involved in a further 11 reindeer given the lack of male nematodes, or in two reindeer where the identification was limited to *Ostertagia* sp. due to degradation of the sample which made it impossible to visualise the structures clearly.

## 4 Discussion

### 4.1 Parasite diversity, methodological challenges and biases

*Spiculopteragia boehmi* and one individual parasite was observed that appeared consistent with *Ostertagia gruehneri's* minor morphotype *O. arctica,* were detected for the first time in this population. Both *O. gruehneri* and *S. boehmi* have been reported in reindeer from North America whilst *O. gruehneri/O. arctica* as well as *S. boehmi/S. mathevossiani* and *S. dagestanica* have been identified in reindeer in Norway (De Bruyn, 2010; Eira et al., 2025; Fruetel and Lankester, 1989; Lichtenfels et al., 1990; Lichtenfels and Hoberg, 1993; Tømterud, 2025). Molecular analysis supports *S. boehmi* having dimorphic males with *S. mathevossiani* being the minor morph (Liénard et al., 2006). As Icelandic reindeer descend from Norwegian semi-domesticated reindeer, O. *gruehneri/O. arctica* and S. *boehmi* have likely sustained their life cycles in Iceland, similar to the *Eimeria* species described first in Iceland, and later found in Norway (Guðmundsdóttir and Skírnisson, 2005; Idland et al., 2021; Yakimoff et al., 1936). The overabundance of *T*. *circumcincta* complex in the reindeer abomasa highlights the spill-over of sheep parasites into this reindeer population. It is also possible that some reindeer parasites, like *O. gruehneri*, could spill over from reindeer to sheep (Manninen et al., 2014), and it would be interesting to investigate to what degree reindeer parasites can be detected in sheep in areas of sympatric grazing in Iceland.

The prolonged storage and transport time under unstable temperature conditions degraded sample quality so that morphological identification of the female nematodes via evaluation of the cuticular ridge patterns was not possible and many of the male *Ostertagia* sp. could not be identified beyond genus level. Further confirmation is therefore needed, especially given that only a single specimen of probable *O. arctica* was identified, and the use of lactophenol precluded further molecular identification. For optimal morphological identification, the abomasal contents should be examined fresh, or cryopreservants like glycerol should be used prior to freezing (Beraldo and Pascotto, 2014). Molecular screening could confirm rare or degraded specimens as well as open for the exploration of faecal nemabiomes (González et al., 2023; Halvarsson et al., 2022). Differentiating the males in polymorphic species would still require morphological analysis, therefore a combined approach would be optimal.

Overall, the mean number of abomasal adult nematodes and the prevalence of these was lower in our study but not significantly different from those previously reported (Guðmundsdóttir, 2006) who identified *T. circumcincta, O. ostertagi* and *T. axei*. There were differences in parasite diversity between the studies which can be explained by small sample sizes, sub-sampling of abomasal contents making it more challenging to detect less prevalent and abundant nematodes, methodological differences (sampling months, volumes examined and fresh versus frozen samples, identification of both male and female nematodes; abomasal digests). Our study did not detect *T. axei*, previously the most prevalent and abundant nematode (Guðmundsdóttir, 2006). Guðmundsdóttir reported that *T. axei* had a higher winter than summer prevalence and abundance, whilst the opposite trend was seen with *T. circumcincta* and a continuous year-round, albeit very low prevalence of *O. ostertagi*. Our study did not investigate winter prevalence nor were abomasal digests carried out. The intraepithelial location of *T. axei* (Grant, 2015) means that it attaches deeply to the abomasal mucosa so washing alone underestimates adult *T. axei* abundance (Gaba et al., 2006; Vignau et al., 2005) and counts should be based on tissue digestion methods. This could explain why we did not detect *T. axei* in our study. However, seasonal differences or the possibility that this parasite was amongst those recovered from the ten reindeer in which species-level identification was not possible, cannot be ruled out.

### 4.2 Trends in prevalence and abundance

#### 4.2.1 Geographic differences in Iceland and between reindeer from Iceland and Norway

Geographic differences in parasite egg output were observed, with reindeer from the East showing higher mean Trichostrongylidae EPG abundance, despite similar abomasal nematode abundance across all management areas. Further investigation is needed to understand why Trichostrongylidae EPG abundance varies geographically, even though prevalence and intensity do not show significant differences, since abundance represents a composite metric integrating these two parameters. The animals from the East region had both the highest Trichostrongylidae egg prevalence and mean intensity, albeit not significantly higher, which is in part a reflection of the smaller samples sizes when comparing only the infected population in intensity data. Higher abundance may reflect differences in reindeer sex ratios, stress-related immune responses, or abomasal and intestinal Trichostrongylidae species composition, as some species are more fecund than others (Taylor et al., 2016). However, it could also be related to local climatic, vegetation and faunal (co-grazing animals) differences impacting parasite transmission and prevalence. Locally higher sheep densities are reported in the eastern fjords (>10 sheep/km2; Árnason et al., 2022) than in the other regions. The geographic difference in abundance EPG is, however, most likely due to the skewed reindeer sex distribution since statistical modelling, after all, identified sex, rather than geographic origin, as significant explanatory factors for Trichostrongylidae egg abundance and proportionately more males were sampled in the East management area. Male reindeer had, after all, significantly higher Trichostrongylidae egg prevalence and abundance than females.

Comparison of prevalence, abundance and diversity between Iceland and Norway (Table 1) showed higher Trichostrongylidae egg prevalence in five of the seven Norwegian herds (four semi-domesticated and three wild) investigated and in one for *Aonchotheca* (Eira, 2022; Guðmundsdóttir, 2006; Idland et al., 2021; Utaaker et al., 2023). The mean egg abundance (EPG) in the Norwegian reindeer was higher than the upper 95 % CI limit for the Icelandic reindeer, for both types of eggs in all four of the semi-domesticated herds. Whilst two of three wild reindeer herds also had significantly higher mean Trichostrongylidae and *Aonchotheca* sp. EPG. The Norwegian herds also showed greater parasite egg species diversity with more generalist nematode species detected. This indicates that the infection pressure for Icelandic reindeer remains low and that the infection pressure from co-grazing sheep in Iceland is probably lower than in Norway. Sheep densities in the reindeer areas in Iceland (∼5.5 sheep/km^2^), are lower than in the wild reindeer areas investigated in Norway (∼13–16 sheep/km^2^) (Árnason et al., 2022; þórisson et al., 2021). Skírnisson (2011) detected Trichostrongylidae eggs (mean EPG 500) in lambs returning from summer pastures, confirming that upland transmission of Trichostrongylidae occurs throughout summer in this region. Gastrointestinal nematodes, including Nematodirinae, *Trichuris ovis*, *T. circumcincta*, *T. capricola*, *T. vitrinus*, *T. axei*, *Chabertia ovina* and *Oesophagostomum venulosum*, have been detected in sheep in Eastern Iceland (Richter, 2002). These might also be expected to be found in reindeer. A study investigating parasite infection dynamics in ewe lambs from Eastern Iceland, from round-up in September until the following autumn, showed that infection with *Trichuris* and Nematodirinae occurred during the autumn after their return from summer upland grazing to the lowland farms whilst ewes returning the following summer only had low Nematodirinae infection burdens (Skírnisson, 2011). This could partly explain why we are not seeing spillover to reindeer with these nematodes.

#### 4.2.2 Differences between age and sex

Age and sex influenced parasite patterns. These findings suggest complex host-parasite interactions that warrant further investigation. Reindeer calves had a significantly higher prevalence of Trichostrongylidae eggs in 2018 compared to 2003. No differences were seen between the other age classes. The calf samples in 2003 were from faecal scats collected from the ground rather than directly from the animals and therefore hatching of the Trichostrongylidae eggs could have potentially occurred in the environment prior to collection and analysis. There were no significant differences in mean egg abundance by age class for either Trichostrongylidae or *Aonchotheca* sp. between the two study periods, which would suggest that hatching alone does not explain the difference in prevalence. This difference could therefore be due to slightly later sampling dates in 2018 compared to 2003. Calves, in 2018, would have had an additional month of grazing in which to develop patent parasite infections. The *Aonchotheca* prevalence was significantly higher in 2018 than 2003, although no differences were seen between individual age classes. Temporal differences in sampling would need to be further investigated to confirm if there is a true increase in faecal egg counts from mid to late summer or annual fluctuations.

Female reindeer had lower Trichonstrongylidae egg prevalence and abundance counts, than their male counterparts during autumn sampling in 2018. This sex difference has been seen in other cervid species (Davidson et al., 2015; Irvine et al., 2006). These studies hypothesised that males have larger abomasal volumes and more space for nematodes and thus higher egg counts. In our dataset the males did have significantly higher abomasal counts in non-parametric analyses, which might support this. However, alternative explanations include reduced feed intake close to the rut in males leading to higher apparent faecal egg counts (Stear et al., 1995; Stien et al., 2002; Wilson et al., 2002). Another explanation is reproduction-parasitism trade-offs (Malo et al., 2009; Sheldon and Verhulst, 1996) with high testosterone levels prior to the rut having a negative impact on host-immunity, resulting in higher levels of parasitism. The significant correlation between egg abundance and abomasal nematode burden in males, and the lack of it in females, could support the relaxed immunity hypothesis. Interestingly reindeer brought indoors during experimental work, and under increased stress, had a five-fold jump in Trichostrongylidae egg output between day zero and seven (Davidson et al., 2023). Stress, without additional infection pressure, can therefore result in dramatic acute increases in egg output. Different levels of hunting pressure on the different sexes and between management areas might also result in differences in stress levels. Differences in stress between reindeer from the different management areas was however not measured as part of this study. The higher EPG in males in autumn (close to the rutting season), is probably multifactorial. Teasing apart the different contributing factors requires more detailed investigation (Albery et al., 2019).

### 4.3 Conclusion

In conclusion, reindeer in Iceland continue to have low abomasal nematode counts, dominated by *T. circumcincta* and a low abundance and intensity of gastrointestinal parasite eggs. The detection of *S. boehmi* and the possible identification of *O. gruehneri,* highlight the challenges of detecting less prevalent species in small sub-samples. Future work should try to investigate fresh abomasal contents, or when this is not possible include the addition of cryo-preservatives prior to freezing, for improved morphological evaluation. Further morphological or molecular confirmation is needed to determine with certainty the different *Ostertagia* species in this isolated reindeer population. The changing climate may impact the resilience of this population and their parasite burdens even though many of the reindeer specialised climate sensitive parasites, like warble flies (*Hypoderma tarandi*), microfilaria such as the peritoneal worm (*Setaria tundra*), and brainworm (*Elaphostrongylus rangiferi*), are not present in this population.

## CRediT authorship contribution statement

**R.K. Davidson:** Writing – review & editing, Writing – original draft, Visualization, Supervision, Resources, Methodology, Investigation, Formal analysis, Data curation. **S. Dembereldagva:** Writing – review & editing, Writing – original draft, Visualization, Investigation, Formal analysis, Data curation. **I.H. Nymo:** Writing – review & editing, Resources, Investigation, Funding acquisition. **T. Mørk:** Writing – review & editing, Resources, Investigation. **J. Sánchez Romano:** Writing – review & editing, Resources, Investigation. **R. þórarinsdóttir:** Writing – review & editing, Investigation, Conceptualization. **K.S. Utaaker:** Writing – review & editing, Investigation. **S.G. þórisson:** Writing – review & editing, Funding acquisition, Conceptualization. **M. Tryland:** Writing – review & editing, Supervision, Resources, Project administration, Methodology, Investigation, Funding acquisition, Conceptualization.

## Declaration of generative AI and AI-assisted technologies in the writing process

During the preparation of this work the authors used Chat GPT to format the reference list and help edit an image in the graphic abstract. After using this tool/service, the authors reviewed and edited the content as needed and take full responsibility for the content of the publication.

## Funding

The study was funded by grants from FRAM-High North Research Centre for Climate and the Environment, through the project « 10.13039/100018696Health and infectious diseases in semi-domesticated reindeer in a changing climate» (Grant no. 362256) and by grants from the project: Climate-change effects on the epidemiology of infectious diseases and the impacts on Northern Societies (CLINF; www.clinf.org), funded by 10.13039/501100004785NordForsk (Grant no. 76413).

## Declaration of competing interest

The authors confirm they have no conflicts of interest.
